# Highly Dispersed Ag_2_S Nanoparticles: In Situ Synthesis, Size Control, and Modification to Mechanical and Tribological Properties towards Nanocomposite Coatings

**DOI:** 10.3390/nano9091308

**Published:** 2019-09-12

**Authors:** Yanjun Ma, Zhicheng Zhao, Yanbo Xian, Hongqi Wan, Yinping Ye, Lei Chen, Huidi Zhou, Jianmin Chen

**Affiliations:** 1 State Key Laboratory of Solid Lubrication, Lanzhou Institute of Chemical Physics, Chinese Academy of Sciences, Lanzhou 730000, China; mayanjun@licp.cas.cn (Y.M.); zczhao17@licp.cas.cn (Z.Z.); xianyanbo99@163.com (Y.X.); chenlei@lzb.ac.cn (L.C.); hdzhou@lzb.ac.cn (H.Z.); 2 Center of Materials Science and Optoelectronics Engineering, University of Chinese Academy of Sciences, Beijing 100049, China; 3 Department of Materials Science and Engineering, Lanzhou University of Technology, Lanzhou 730050, China

**Keywords:** in situ synthesis, size control, Ag_2_S nanoparticles, mechanical properties, tribological properties, nanocomposite coating

## Abstract

A facile in situ synthesis approach and a size control strategy were established to obtain Ag_2_S nanoparticles in polyimide (PI) composite coatings. Such Ag_2_S nanoparticles in the composite coatings were characterized, and the effects of the as-obtained Ag_2_S nanoparticles of different sizes on the mechanical and tribological properties of the nanocomposite coatings were investigated. Results indicate that the in situ synthesized Ag_2_S nanoparticles exhibited good dispersibility and bimodal and multimodal size distribution in the nanocomposite coatings. The size of the Ag_2_S nanoparticles can be effectively controlled by adjusting the substituent alkyl chain length of single-source precursor, and these Ag_2_S nanoparticles exhibited superior improvement to mechanical and tribological properties of the nanocomposite coatings. More importantly, the Ag_2_S nanoparticles with the proper grain size and bimodal size distribution provided the optimal mechanical and tribological properties for the nanocomposite coatings, and the excellent tribological properties were attributed to their outstanding mechanical properties and strong ability to form a homogenous and stable protective tribofilm.

## 1. Introduction

As functionalizing and reinforcing additives, nanoparticles have exerted a tremendous fascination in composite materials, due to their small size effect and strong interfacial activity with the reinforced matrices [1,2,3,4,5]. Unfortunately, nanoparticles with a high surface energy usually tend to agglomerate, which is hurtful to their dispersion in polymer matrices and to their reinforcing efficacy as well [6,7]. To overcome this drawback, researchers have attempted to improve the dispersibility of nanoparticles through mechanical grinding [8], ultrasonic dispersing [9,10], chemical modification [11,12], and the like. However, these strategies still cannot avoid the re-agglomeration of nanoparticles during the preparation process of composite materials, and it is extremely difficult to obtain the mono-dispersal of nanoparticles in solid materials.

Aside from homogenous dispersion, a variety of other factors, including shape, size, size distribution, and the interaction between nanoparticles and the host matrix, also have significant influences on the reinforcing effect of nanoparticles. It has been found that the optical [13], electrical [14], thermal [15], magnetic [16], antimicrobial [17], catalytic [18], and photovoltaic [19] properties of nanoparticles are greatly influenced by their size. Particularly in the field of tribology, Kalyani et al. [20] and Peng et al. [21] reported that small size nanoparticles as the lubricant additives of paraffin oil can effectively improve the load-bearing capacity and tribological properties. However, Zin et al. [22] argued that large nanoparticles are more efficient in reducing the friction coefficient, probably due to their replenishment effect towards the sliding surface and the roll-bearing effect during the sliding process. Xu et al. [23] said that the bimodal nanoparticle size distribution strategy is also applicable to designing materials with high strength and good wear-resistance, and Echavarria et al. [24] suggested that the desired balance between the mechanical and tribological properties of composite coatings can be achieved by properly adjusting the size of nanoparticles as the lubricant additives. To date, nevertheless, the influence of nanoparticle size and size distribution on their comprehensive properties still waits to be revealed at depth.

As a typical quasi-two-dimensional material, nanostructured silver sulfide (Ag_2_S) is technologically and fundamentally intriguing due to its negligible toxicity, simple synthesis process, high chemical stability, unique layered structure, and the adjustability of its physical and chemical properties by varying the shape and size [25,26,27]. Besides, much attention has been focused on great potentials of Ag_2_S nanoparticles in the field of tribology because of their weak Van Der Waals interaction between the adjacent layers [28]. Many attempts have been made to prepare Ag_2_S nanoparticles in liquid phase. However, the rate of nucleation and growth of Ag_2_S nanocrystal is very fast due to the ultralow solubility product (*K*_sp_ = 6.3 × 10^−50^). As a result, the small size preparation and size control of Ag_2_S nanoparticles have always been a great challenge [29]. Therefore, more effective strategies for preparing small and size-tunable Ag_2_S nanoparticles should be explored for their in-depth research and application.

With the assistance of the in situ thermal decomposition of a single-source precursor, here we establish a facile approach for preparing highly dispersed Ag_2_S nanoparticles in composite materials. By properly adjusting the substituent alkyl chain length of the single-source precursor, we attempt to manipulate the size, shape, and size distribution of the as-prepared Ag_2_S nanoparticles. This article deals with the fabrication of the highly dispersed Ag_2_S nanoparticles as well as the effects of their size on the mechanical and tribological properties of the polyimide (PI) nanocomposite coatings.

## 2. Materials and Methods

### 2.1. Materials

Diethylamine, dibutylamine, dihexylamine, carbon disulfide, and silver nitrate (99.8%) were purchased from Sigma-Aldrich Trading Company Limited (Shanghai, China). Polyamic acid with a solid content of 38.0% was obtained from Beijing Sino-rich Technology Company Limited (Beijing, China). The mixed solvent of N,N-dimethylformamide and N-methylpyrrolidone (volume ratio 1:1) was used as the co-dispersion medium of the paint. All chemicals were directly used as-received. AISI-1045 steel block (19.00 mm × 12.00 mm × 12.00 mm, Rockwellhardness (*HRC*) 28–32, *E* = 210 GPa) was used as the substrate, and AISI-52100 bearing steel ball (*Φ* 6 mm, *HRC* 58–61, *E* = 208 GPa, *R*a = 0.02 μm) was used as the counterpart.

### 2.2. Controllable In Situ Synthesis of Ag_2_S Nanoparticles

**Figure 1:**
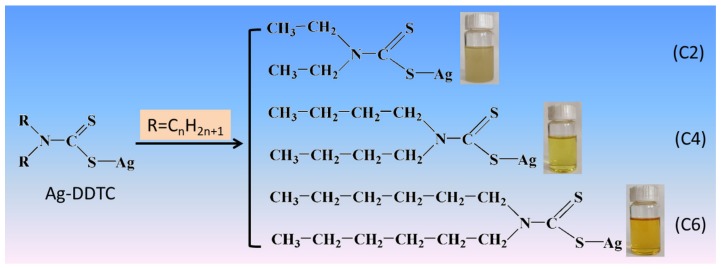
Structural diagram of the single-source precursors.

The preparation of single-source precursor of N,N-dialkyldithiocarbamate silver (DDTC-Ag) was carried out as follows. In a typical preparation procedure of N,N-diethyldithiocarbamate silver, 0.1 M diethylamine and 0.1 M potassium hydroxide were dissolved in 100 mL (95%) ethanol and placed into an ice water bath, followed by the slow addition of 0.12 M carbon disulfide under overnight vigorous stirring at room temperature. The aqueous solution of silver nitrate with an equal stoichiometry was then dropwise added to the resultant solution and stirred for 3 h. The solid component of the precursor was obtained via filtering, washing alternately with ethanol and water, and drying in vacuum oven at 60 °C. A series of single-source precursors were obtained, with the amines possessing different substituent alkyl chain lengths (C_2_H_5_, C_4_H_9_, and C_6_H_13_); and the resultant precursors were denoted as C2, C4, and C6, respectively. The structure of the corresponding single-source precursors are shown in Figure 1.

The as-obtained single-source precursors were separately mixed with polyamic acid uniformly at the same ratio and coated onto the substrate to obtain the wet films (prior to coating, the substrates were sandblasted and cleaned with acetone). The wet films were sequentially cured in a curing chamber at 150 °C for 30 min and at 240 °C for 120 min to obtain the nanocomposite coatings denoted as S-C2, S-C4, and S-C6, and the coating without any nanoparticles, denoted as S-C0, was also prepared under the same conditions and used for comparative studies. It should be noted that the theoretical amount of the in situ synthesized Ag_2_S nanoparticles was uniformly 5.0 wt.% in the PI nanocomposite coatings in this work.

The phase structure of the as-obtained Ag_2_S nanoparticles was analyzed with an X-ray diffractometer (XRD, EMPYREAN, Almelo, The Netherlands; Cu *K*α radiation *λ* = 0.15406 nm) under a voltage of 40 kV and a current of 30 mA. The diffraction angle (2 *θ*) ranged from 5° to 70°, with a step size of 0.1° and time per step of 0.6 s. The size and morphologies of the Ag_2_S nanoparticles were observed with an ultra-high resolution field emission scanning electron microscope (FESEM, Hitachi SU8020, Tokyo, Japan).

### 2.3. Evaluation of Mechanical Properties

The micro-hardness of the nanocomposite coatings was measured with an MH-5-VM type hardness tester (MH-5-VM, China) under a loading force of 10 g and dwell time of 5 s. Six repeating measurements were conducted at different locations for each sample, and the average value was calculated and reported here. The elastic modulus was measured with a CSM indenter (NHT2, Canton, Switzerland) equipped with a Berkovich diamond probe tip, and the indentation tests were conducted under a load of 0.1~10 mN and pause time of 10 s. The scratch test was conducted with a CSM scratch tester (RST^3^, Canton, Switzerland) equipped with a diamond tip (conical angle: 150°) under a maximum load of 20 N and a scratching rate of 10 N/min. Upon completion of the scratch tests, the synchronized panorama images of the scratch tracks were taken with an optical camera. Dynamic mechanical analysis (DMA) was conducted with a dynamic mechanical analyzer (NETZSCH DMA242E, Serb, Germany) under a heating rate of 5 °C/min, vibration frequency of 1 Hz, and temperature ranging from 30 °C to 270 °C. Prior to the DMA measurements, the specimens were cut into strips with dimensions of 21.0 mm × 3.0 mm × 0.4 mm.

### 2.4. Friction and Wear Test

The tribological properties of the as-prepared nanocomposite coatings were evaluated with a CSM ball-on-disk test rig (Tribo-S-D-0000, Canton, Switzerland) under dry sliding conditions (about 25 °C, 30–45% relative humidity). The reciprocal sliding tests were run under an oscillation amplitude of 2.5 mm, a sliding frequency of 7 Hz, an applied load of 5 N, and a sliding distance of 140 m. The friction coefficient was recorded in real time with a gauge affiliated to the tribometer. The wear rate (*W*, unit: mm^3^·N^−1^·m^−1^) was calculated as *W = V_L_/(N·S)*, where *V_L_* (unit: mm^3^) refers to the wear volume measured with a non-contact surface mapping profiler (Micro XAM-3D, ADE Corporation, Washington, USA), *N* (unit: N) is the applied load, and *S* (unit: m) is the total stroke length. At least six repeating measurements were conducted for each specimen under the same conditions in order to minimize scattering. The average friction coefficient and wear rate of the repeating measurements are cited here. The worn morphologies of the wear tracks of the nanocomposite coatings and corresponding counterpart balls were observed with an Olympus optical microscope (MM6C-RS1, Tokyo, Japan). The cross section of the transfer film formed on the counterpart steel ball was obtained with a focused ion beam (FIB), and their morphologies were observed with a transmission electron microscope (TEM, TF20, Oregon, USA).

## 3. Results and Discussion

### 3.1. Size and Morphology of the In Situ Synthesized Ag_2_S Nanoparticles

**Figure 2:**
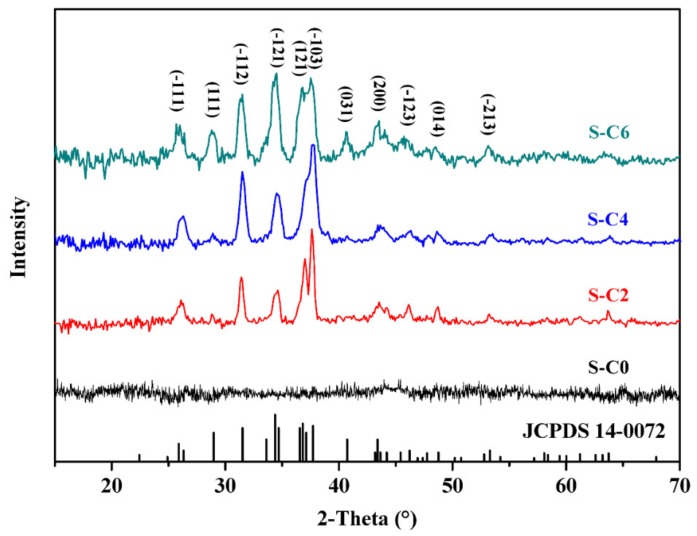
X ray diffraction (XRD) patterns of the nanocomposite coatings with in situ synthesized Ag_2_S nanoparticles from single-source precursors with different substituent alkyl chain length.

The XRD patterns of the nanocomposite coatings containing Ag_2_S nanoparticles made from the precursors with different substituent alkyl chain length are shown in Figure 2. S-C0 did not show apparent diffraction peaks, which corresponds to the low crystallinity of the neat polymer matrices. The Ag_2_S nanoparticles obtained from the precursors with different substituent alkyl chain length in nanocomposite coatings showed diffraction peaks of (−111), (111), (−112), (−121), (121), (−103), (031), (200), (−123), (014), and (−213) crystalline planes, and these diffraction peaks matched well with the characteristic peaks of monoclinic Ag_2_S phase with good crystallinity (standard library card of JCPDS 14-0072, lattice parameter *a* = 4.229, *b* = 6.931, *c* = 7.862) [30,31]. Besides this, the as-prepared Ag_2_S nanoparticles did not show any noticeable peaks of other phases, which shows their high purity. Moreover, as the substituent alkyl chain length of the precursor increased, the diffraction peaks of Ag_2_S nanoparticles tended to broaden, which is because the average grain size of the Ag_2_S nanoparticles tends to decline with increasing substituent alkyl chain length of the precursors [32]. Particularly, compared to the sample of S-C2, the diffraction peaks of samples S-C4 and S-C6 were significantly broadened, so the reduction of average grain size of the Ag_2_S nanoparticles was more pronounced. Several diffraction peaks of crystal plane for sample of S-C6, such as (111), (−121), (121), (−103), were slightly broadened compared to the sample S-C4. This indicates that the substituent alkyl chain length of the precursors had a great influence on the grain size of the in situ synthesized Ag_2_S nanoparticles.

**Figure 3:**
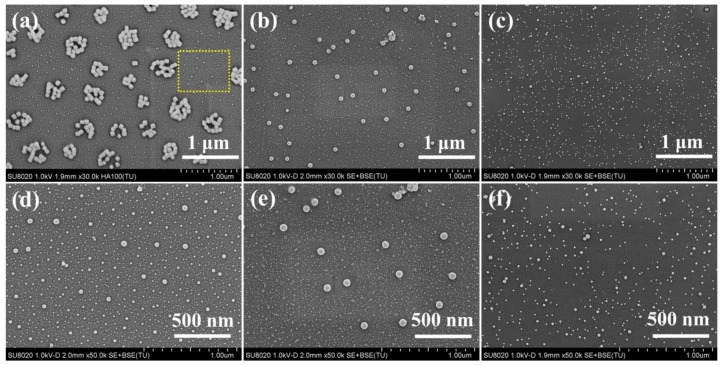
Field emission scanning electron microscope (FESEM) images of Ag_2_S nanoparticles obtained from the single-source precursors with different alkyl chain lengths in the nanocomposite coatings, (**a**) S-C2, (**b**) S-C4, and (**c**) S-C6, as well as corresponding magnified images (**d**–**f**).

FESEM analyses were conducted to further observe the size, morphologies, and dispersion of the in situ synthesized Ag_2_S nanoparticles in the nanocomposite coatings. As shown in Figure 3, the Ag_2_S nanoparticles obtained from precursors with a short substituent alkyl chain exhibited a larger size and multimodal distribution. The large Ag_2_S nanoparticles showed a hexahedral structure and their size was about 90 nm in the nanocomposite coating of S-C2. Interestingly, these nanoparticles had an isolated “island-like” distribution and consisted of aggregates, which could be a result of the weak compatibility between the precursors with a short alkyl chain and the resin matrix, and the low steric hindrance of the short alkyl chain. The magnified image of the yellow area in Figure 3a is shown in Figure 3d. It can be seen that the different sizes ranging from 12 nm to 63 nm of Ag_2_S nanoparticles were presented in the present coating. These observations indicate that the Ag_2_S nanoparticles in the S-C2 nanocomposite coating exhibited a non-uniform morphology and size. As the substituent alkyl chain length of the precursor increased, the as-obtained Ag_2_S nanoparticles showed sphere-like without hexahedron morphology (Figure 3b,e). Particularly, the Ag_2_S nanoparticles in the nanocomposite coating S-C4 were completely isolated and exhibited a bimodal distribution. It is worth noting that the size and morphologies of these bimodal Ag_2_S nanoparticles were very uniform at their respective scale. The larger Ag_2_S nanoparticles were about 75 nm, and the smaller Ag_2_S nanoparticles were about 12 nm, and the numbers of small nanoparticles were much more than that of the large nanoparticles, which can possibly be attributed to the increasing compatibility of the precursor C4 with a long alkyl chain with the resin matrix. Moreover, the longer alkyl chain provided greater steric hindrance, which also effectively blocked the contact and collision between the molecules, whether precursor or nanocrystalline, resulting in smaller nanoparticles. As the substituent alkyl chain length of the precursor further increased, the size of the as-obtained Ag_2_S nanoparticles in the nanocomposite coating S-C6 was reduced and their size distribution was multimodal (from 9 nm to 30 nm, Figure 3c,f), and these Ag_2_S nanoparticles were still well-dispersed in the nanocomposite coating, without any agglomeration. This result can be understood by the fact that the longer-chain precursor molecule provided better compatibility and better dispersion (precursor solution is transparent) in the matrix, however, organic molecules with excess chain length will entangle each other, resulting in a non-uniform size of the resulting Ag_2_S nanoparticles. This indicates that the substituent alkyl chain length of the precursor greatly affects the size and size distribution of the as-prepared Ag_2_S nanoparticles, and it is feasible to manipulate the size and size distribution of the Ag_2_S nanoparticles by adjusting the substituent alkyl chain length of the precursor.

### 3.2. Effect of Ag_2_S Nanoparticles on Mechanical Properties of Nanocomposite Coatings

**Figure 4:**
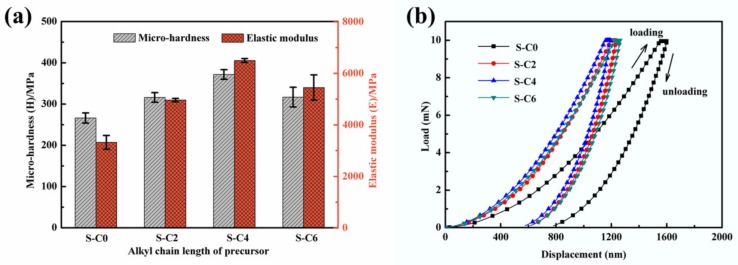
Effect of the in situ synthesized Ag_2_S nanoparticles on mechanical properties of the nanocomposite coatings, (**a**) micro-hardness and elastic modulus, (**b**) load-displacement curves.

The micro-hardness, elastic modulus, and load-displacement curves of the nanocomposite coatings are presented in Figure 4. It can be seen that the incorporation of Ag_2_S nanoparticles resulted in an increase of the micro-hardness and elastic modulus of the nanocomposite coatings (Figure 4a), and in particular, the nanocomposite coating S-C4 had the maximum micro-hardness (372 MPa) and elastic modulus (6497 MPa). Similarly, the in situ synthesized Ag_2_S nanoparticles contributed to decreasing the critical displacement (i.e., indentation depth) and residual depth of the nanocomposite coatings (Figure 4b). Namely, S-C0 without Ag_2_S nanoparticles had a maximum displacement of 1600.35 nm and residual depth of 780.16 nm, while S-C4 with incorporated Ag_2_S nanoparticles had a minimum displacement of 1192.20 nm and residual depth of 575.44 nm. This indicates that the incorporation of the in situ synthesized Ag_2_S nanoparticles, especially obtained from the precursor of C4, contributed to increasing the resistance to plastic deformation and elastic recovery ability of the nanocomposite coatings [33,34,35].

**Figure 5:**
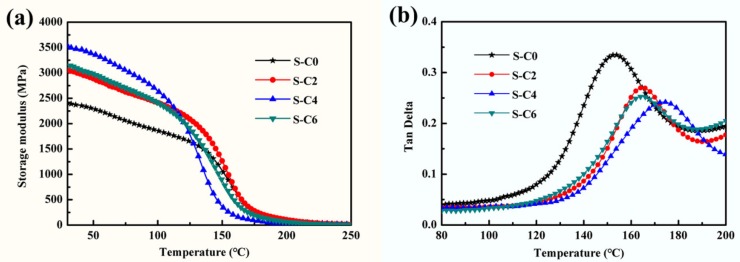
Dynamic thermal mechanical properties of the nanocomposite coatings with in situ synthesized Ag_2_S nanoparticles, (**a**) storage modulus, (**b**) loss factor.

The effects of the incorporated in situ synthesized Ag_2_S nanoparticles on the dynamic thermal mechanical properties of the nanocomposite coatings are shown in Figure 5. The storage modulus in the glassy regions, representing the stiffness, tended to increase after the introduction of the in situ generated Ag_2_S nanoparticles (Figure 5a), and in particular, the nanocomposite coating S-C4 exhibited the highest storage modulus (referring to the highest load-bearing capacity), which corresponds well with its high micro-hardness and elastic modulus [36,37,38]. In addition, the peak location in loss factor (tan*δ*)–temperature curve represents the glass transition temperature of the material, and the peak intensity reflects the ability to dissipate energy [39]. As can be seen from Figure 5b, the glass transition temperature of the pure polymer resin was 153 °C, which increased for nanocomposite coatings with in situ generated Ag_2_S nanoparticles. Among them, the glass transition temperature of nanocomposite coating SC4 was increased to 175 °C. The peak intensity of the tan*δ* curves of the nanocomposite coatings with in situ synthesized Ag_2_S nanoparticles stayed below that of the polymer matrix, and S-C4 exhibited lowest value of loss factor, indicating its high ability to attenuate the internal energy dissipation in association with damping characteristics [40], which can be attributed to a higher degree of crosslinking and increased homogeneity as well [41]. All of these results indicate that the incorporation of in situ synthesized Ag_2_S nanoparticles significantly enhanced the dynamic thermomechanical properties of the nanocomposite coatings.

**Figure 6:**
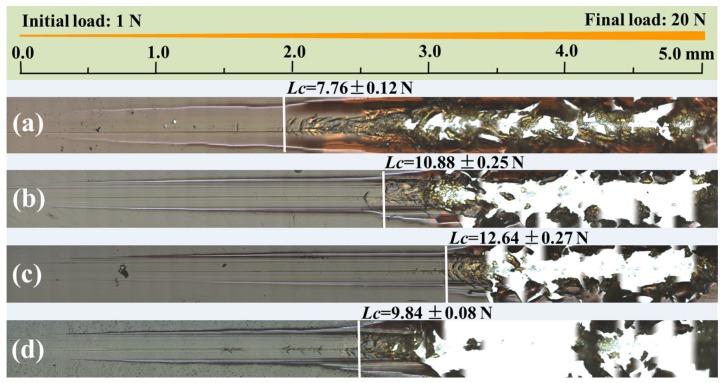
Panorama images of the blank polymer matrix and the nanocomposite coatings after scratch testing, (**a**) S-C0, (**b**) S-C2, (**c**) S-C4, and (**d**) S-C6.

Figure 6 shows the panorama images of the blank polymer matrix and the nanocomposite coatings after scratch tests. The scratch resistance was evaluated with the critical load (*L*c) where cracks initiated. The neat polymer matrix, S-C0 without Ag_2_S nanoparticles, underwent damage at a low *L*c of 7.76 ± 0.12 N in the scratch test (Figure 6a). After the incorporation of Ag_2_S nanoparticles, the failure of the nanocomposite coatings during the scratch tests occurred at increased critical loads, and in particular, the nanocomposite coating S-C4 exhibited a maximum *L*c value of 12.64 ± 0.27 N (Figure 6c), much higher than that of the neat polymer matrix. This indicates that the nanocomposite coating S-C4 exhibits excellent bonding strength and internal strength [33]. Therefore, the precursor C4 was suggested to be adopted for fabricating nanocomposite coating S-C4, with uniformly dispersed and well-bonded Ag_2_S nanoparticles, so as to more effectively improve the mechanical properties of nanocomposite coatings. At the same time, it could be rationally believed that the enhancement effect of nanoparticles for a polymer matrix depends not only on their particle size but also on their size homogeneity.

The representative numerical parameters extracted from the mechanical tests of the nanocomposite coatings are summarized in Table 1. From the results of mechanical tests, we can conclude that the incorporation of in situ synthesized Ag_2_S nanoparticles significantly improved the mechanical properties of the nanocomposite coatings. More importantly, the improvement of Ag_2_S nanoparticles to the mechanical properties of nanocomposite coatings depended not only on their dispersibility but also on their size and size distribution. The in situ generated Ag_2_S nanoparticles exhibited a larger size and a disordered size distribution in the nanocomposite coating of S-C2, and the partial nanoparticles underwent an island-like agglomeration, which greatly limited their reinforcing effect on the mechanical properties of nanocomposite coatings. In the nanocomposite coating of S-C4, the Ag_2_S nanoparticles with a good dispersibility and a bimodal distribution provided good homogeneity for the nanocomposite coating, which alleviated the internal stress caused by volume shrinkage during the curing process of the coating to a large extent. In addition, the larger Ag_2_S nanoparticles provided a stronger supporting effect for the nanocomposite coating, and the smaller particles further synergistically maximized the reinforcing effect. In this case, the high strength was achieved by the bimodal grain size distribution. Therefore, the nanocomposite coating of S-C4 exhibited the optimal mechanical properties. Despite obtaining good dispersion, the smaller Ag_2_S nanoparticles with multimodal distribution disturbed the homogeneity of the nanocomposite coating of S-C6 and did not provide effective support, which may result in the attenuation of their mechanical properties.

### 3.3. Effect of Ag_2_S Nanoparticles on Tribological Properties of Nanocomposite Coatings

**Figure 7:**
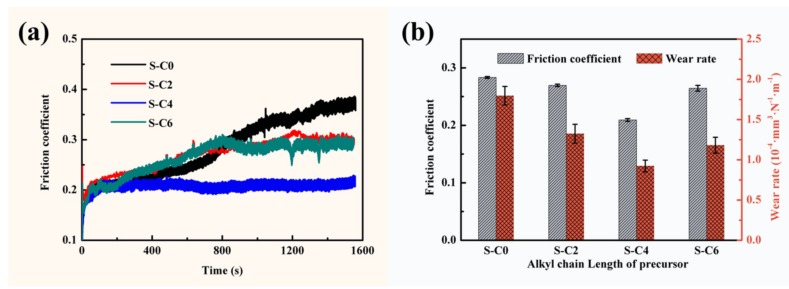
Friction coefficient and wear rate of the nanocomposite coatings, (**a**) kinetic curves of the friction coefficient, (**b**) average friction coefficient and wear rate.

The effect of the grain size of the in situ synthesized Ag_2_S nanoparticles on the friction and wear behavior of the nanocomposite coatings was further explored under dry friction conditions. The friction coefficient and wear rates of the nanocomposite coatings are displayed in Figure 7 and Table 2. As shown in Figure 7, the coating (S-C0) without Ag_2_S nanoparticles exhibited a rather unstable and higher friction coefficient of 0.284. It was delightful to see the positive response to the incorporation of in situ generated Ag_2_S nanoparticles. The friction coefficient of the nanocomposite coatings greatly decreased and dynamic curves became relatively stable compared to that of the blank resin system. In this expected gratifying situation, the nanocomposite coating (S-C4) with Ag_2_S nanoparticles derived from the precursor of C4 showed the most stable and lowest friction coefficient of 0.210 among the four kinds of tested coatings, and the friction coefficients of S-C2 and S-C6 were 0.270 and 0.265, respectively. Besides this, the nanocomposite coating of S-C0 without Ag_2_S nanoparticles had a high wear rate of 1.80 × 10^−4^ mm^3^·N^−1^·m^−1^, and those nanocomposite coatings containing in situ generated Ag_2_S nanoparticles had lowered wear rates. In particular, the nanocomposite coating S-C4 exhibited a minimum wear rate of 9.24 × 10^−5^ mm^3^·N^−1^·m^−1^, which reduced by approximately 47.78% compared to that of S-C0, as well as less than that of S-C2 and S-C6. The wear rate of the nanocomposite coating S-C6 containing in situ synthesized Ag_2_S nanoparticles of a smaller size and non-uniformity was lower than that of S-C2 with larger-sized Ag_2_S nanoparticles. Therefore, it can be concluded from these analyses that the in situ generated Ag_2_S nanoparticles contributed to intensely improving the tribological properties of the nanocomposite coatings, and their reinforcing effect also highly dependent on their small size and size distribution, which was completely consistent with the testing results of mechanical properties.

### 3.4. Analyses of Wear Track Morphology and Wear Mechanism

**Figure 8:**
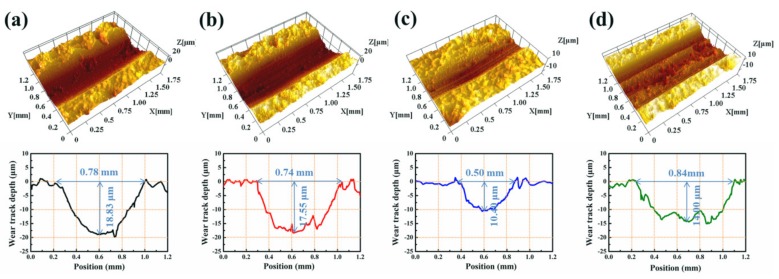
3D morphologies of wear tracks and cross-sections, (**a**) S-C0, (**b**) S-C2, (**c**) S-C4, and (**d**) S-C6.

Figure 8 shows the three-dimensional (3D) morphologies of the worn surfaces and cross-sections of the wear tracks for various nanocomposite coatings. The blank polymer coating without Ag_2_S nanoparticles (S-C0) displayed serious wear damage, and its wear depth and width were as much as 18.83 μm and 0.78 mm, respectively (Figure 8a). After the incorporation of the in situ generated Ag_2_S nanoparticles, the wear tracks of the nanocomposite coatings became shallow and narrow, which corresponds with its enhanced wear resistance. In particular, the wear track depth (10.40 μm) and width (0.50 mm) of the nanocomposite coating S-C4 were the lowest compared with those of S-C2 and S-C6 (Figure 8b–d). These results again demonstrate that the in situ synthesized Ag_2_S nanoparticles in sample S-C4 were more effective in improving the tribological properties of the nanocomposite coating.

**Figure 9:**
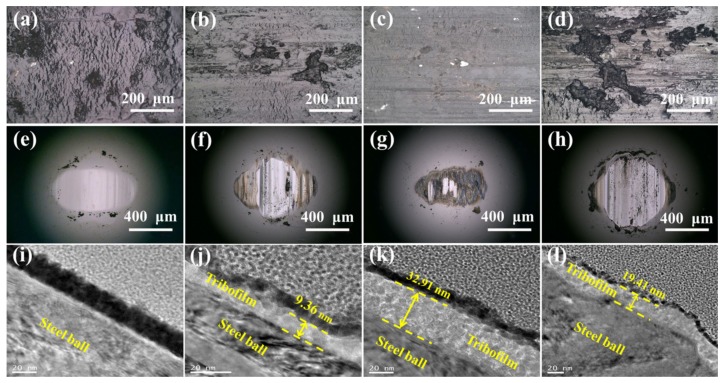
Optical photographs of worn surfaces and wear scars of corresponding counterpart balls sliding against the nanocomposite coatings, as well as transmission electron microscope (TEM) morphologies of tribofilm, (**a**,**e**,**i**) S-C0, (**b**,**f**,**j**) S-C2, (**c**,**g**,**k**) S-C4, (**d**,**h**,**l**) S-C6.

For clarifying the wear mechanism of the nanocomposite coatings, Figure 9 shows the worn morphologies of the coating surfaces and the counterpart ball, as well as the transmission electron microscope (TEM) morphologies of the transfer film on the counterpart ball. As shown in Figure 9a, the worn surface of sample S-C0 showed signs of severe damage accompanied by a lot of large cracks, and the material was detached after the cracks connected with each other. The wear domain of the corresponding counterpart steel ball showed a large area (Figure 9e), and there was no transfer film formed on the counterpart steel ball (Figure 9i). Once Ag_2_S nanoparticles were introduced, the worn damage of the coating surfaces was significantly alleviated. For the nanocomposite coating of S-C2 (Figure 9b) and S-C6 (Figure 9d), the wear defects and cracks on the worn surface were suppressed, and the wear area of the corresponding counterpart ball (Figure 9f,h) was reduced. The transfer films of 9.36 nm for S-C2 and 19.41 nm for S-C6 were formed on the counterpart steel ball (Figure 9j,l). However, these transfer films were of poor uniformity, allowing partial steel-coating direct contact at some locations, thereby causing wear damage in some areas [42]. In particular, for the nanocomposite coating of S-C4, the worn surface was relatively smooth, with no obvious defects except for some micro-cracks (Figure 9c). The wear area of the corresponding counterpart ball was also significantly reduced (Figure 9g). In addition to the excellent mechanical properties of the S-C4 nanocomposite coating, the improvement in tribological properties was apparently attributed to the formation of the relatively uniform and thick transfer film on the counterpart ball (Figure 9k). These worn surfaces and transfer film morphologies of the coatings and counterpart balls indicate that the Ag_2_S nanoparticles in the nanocomposite coating SC-4 were conducive to forming a homogeneous and stable protective tribofilm, which prevented direct contact between the nanocomposite coating and the counterpart steel ball, thereby playing an effective role in enhancing the wear resistance [43,44,45]. Therefore, it is reasonable to believe that an appropriate grain size and a bimodal size distribution strategy for in situ synthesized Ag_2_S nanoparticles can improve the mechanical and tribological properties of the nanocomposite coatings more effectively.

## 4. Conclusions

Highly dispersed Ag_2_S nanoparticles were successfully synthesized in situ via the thermal decomposition of the N,N-dialkyldithiocarbamate silver in the nanocomposite coatings. The size and size distribution of the Ag_2_S nanoparticles was well manipulated by adjusting the substituent alkyl chain length of the single-source precursor. The incorporated in situ synthesized Ag_2_S nanoparticles significantly improved the mechanical properties (such as micro-hardness, elastic modulus, resistance to plastic deformation, elastic recovery ability, storage modulus, and scratch resistance) and tribological properties of the nanocomposite coatings. In particular, the Ag_2_S nanoparticles with good dispersion, proper grain size, and bimodal size distribution offered the optimal improvement for the mechanical and tribological properties of the nanocomposite coatings, and the excellent tribological properties were attributed to their remarkable mechanical properties and strong ability to form a uniform and stable transfer film.

## Figures and Tables

**Table 1:** Representative parameter values extracted from mechanical tests of nanocomposite coatings.

Samples	S-C0	S-C2	S-C4	S-C6
Micro-hardness (*H*, Mpa)	266	316	372	317
Elastic modulus (*E*, Mpa)	3322	4963	6497	5448
Indentation depth (nm)	1600.35	1233.36	1192.20	1261.70
Storage modulus (*E’*, Mpa)	2423.83	3065.64	3552.48	3143.89
Loss factor (Tan*δ*)	0.3348	0.2704	0.2413	0.2529
Glass transition temperature (*T*_g_, °C)	153	165	175	164
Critical load (*L*_c_, N)	7.76 ± 0.12	10.88 ± 0.25	12.64 ± 0.27	9.84 ± 0.08

**Table 2:** Average friction coefficient and wear rates of nanocomposite coatings.

Samples	S-C0	S-C2	S-C4	S-C6
Average friction coefficient (*µ*)	0.284	0.270	0.210	0.265
Wear rate (*W*, mm^3^·N^−1^·m^−1^)	1.80 × 10^−4^	1.32 × 10^−4^	9.24 × 10^−5^	1.18 × 10^−4^
